# Genetic, metabolic and cellular factors influencing intracellular localization of the Wilson disease protein, ATP7B

**DOI:** 10.1186/1755-8166-7-S1-P68

**Published:** 2014-01-21

**Authors:** Arnab Gupta, Ashima Bhattacharjee, Nesrin Hasan, Lita Braiterman, Svetlana Lutsenko, Ann L Hubbard

**Affiliations:** 1 Department of Cell Biology, Johns Hopkins University, Baltimore, USA; 2 Department of Physiology, Johns Hopkins University, Baltimore, USA

## Background

Wilson disease (WD) is a disorder of copper accumulation in liver and brain caused by mutations in the copper-transporting ATPase ATP7B that affects 1 in 5000 live births. Under basal conditions, ATP7B protein localizes to the trans-Golgi network (TGN) but traffics to vesicles in response to high copper. The purpose of the study is to identify genetic, metabolic and regulatory factors that regulate ATP7B function and localization to maintain normal copper homeostasis in cells. The study is divided into three parts, (a) Role of copper in normal protein folding and its ER exit, (b) Role of regulatory phosphorylation of ATP7B in its trafficking from TGN to vesicles (c) Interaction of ATP7B with regulatory proteins in its trafficking route.

## Material and methods

Recombinant ATP7B (wt and variants), Confocal microscopy, NMR, Mass Spectroscopy, targeted knockdowns and other basic molecular biology techniques were utilized in this study.

## Results

We demonstrate that the polymorphism (producing the Gly^875^>Arg substitution) of ATP7B drastically alters the intracellular properties of ATP7B, while copper reverses the effects. Unlike the wtATP7B, the Arg^875^ variant is located in the endoplasmic reticulum (ER) and does not deliver copper to the TGN. Elevated copper rectifies the ATP7B-Arg^875^ phenotype. Analysis by NMR suggests that the ER retention of ATP7B-Arg^875^ is due to increased unfolding of the Arg^875^-containing.conserved domain (b) I characterized the sites of copper dependent regulatory phosphorylation of ATP7B critical in normal ATP7B trafficking. I discovered that a stretch of Ser (S^340-343^) at the N-terminal of ATP7B are the sites of phosphorylation and that phosphorylation alters the interaction between two conserved domains of ATP7B, causing the protein to attain a TGN exit conformation (c) Finally, while identifying the cellular machinery that is involved in trafficking of ATP7B vesicles, I found that MyosinVb is a major regulator of ATP7B trafficking. Presently, the molecular mechanism of the role of MyoVb in ATP7B trafficking is being investigated.

## Conclusion

We characterized an ATP7B genetic variant (c.2623A/G) that is causal to the disease in Indians and is a non-disease causing SNP in Chinese population.

